# Hepatic cholesteryl ester accumulation in lysosomal acid lipase deficiency: Non-invasive identification and treatment monitoring by magnetic resonance

**DOI:** 10.1016/j.jhep.2013.04.016

**Published:** 2013-09

**Authors:** Peter E. Thelwall, Fiona E. Smith, Mark C. Leavitt, David Canty, Wei Hu, Kieren G. Hollingsworth, Christian Thoma, Michael I. Trenell, Roy Taylor, Joseph V. Rutkowski, Andrew M. Blamire, Anthony G. Quinn

**Affiliations:** 1 Institute of Cellular Medicine, Newcastle University, Newcastle upon Tyne, UK; 2 Newcastle Magnetic Resonance Centre, Newcastle University, Newcastle upon Tyne, UK; 3 Synageva BioPharma Corp., 128 Spring Street, Lexington, MA 02421, USA

**Keywords:** CESD, cholesteryl ester storage disease, LAL, lysosomal acid lipase, CE, cholesteryl ester, TG, triglyceride, ERT, enzyme replacement therapy, NAFLD, non-alcoholic fatty liver disease, Wolman disease, Cholesteryl ester storage disease, ^1^H MR spectroscopy, ^13^C MR spectroscopy, Liver fat, Lysosomal acid lipase, LIPA, LAL deficiency, Enzyme replacement therapy, Sebelipase alfa

## Abstract

**Background & Aims:**

Lysosomal Acid Lipase (LAL) deficiency is a rare metabolic storage disease, caused by a marked reduction in activity of LAL, which leads to accumulation of cholesteryl esters (CE) and triglycerides (TG) in lysosomes in many tissues. We used ^1^H magnetic resonance (MR) spectroscopy to characterize the abnormalities in hepatic lipid content and composition in patients with LAL deficiency, and in *ex vivo* liver tissue from a LAL deficiency rat model. Secondly, we used MR spectroscopy to monitor the effects of an enzyme replacement therapy (ERT), sebelipase alfa (a recombinant human lysosomal acid lipase), on hepatic TG and CE content in the preclinical model.

**Methods:**

Human studies employed cohorts of LAL-deficient patients and NAFLD subjects. Rat experimental groups comprised *ex vivo* liver samples of wild type, NAFLD, LAL-deficient, and LAL-deficient rats receiving 4 weeks of sebelipase alfa treatment. Hepatic ^1^H MR spectroscopy was performed using 3T (human) and 7T (preclinical) MRI scanners to quantify hepatic cholesterol and triglyceride content.

**Results:**

CE accumulation was identified in LAL deficiency in both human and preclinical studies. A significant decrease in hepatic CE was observed in LAL-deficient rats following treatment with sebelipase alfa.

**Conclusions:**

We demonstrate an entirely non-invasive method to identify and quantify the hepatic lipid signature associated with a rare genetic cause of fatty liver. The approach provides a more favorable alternative to repeated biopsy sampling for diagnosis and disease progression / treatment monitoring of patients with LAL deficiency and other disorders characterised by increased free cholesterol and/or cholesteryl esters.

## Introduction

Lysosomal Acid Lipase (LAL) deficiency is a rare autosomal recessive lysosomal storage disease for which there is no currently available effective treatment. The disease is caused by mutations of the *LIPA* gene encoding the LAL enzyme and deficiency of this enzyme leads to the accumulation of cholesteryl esters (CE) and triglycerides (TG) in a number of tissues. Although a single disease, LAL deficiency presents as a clinical continuum with two major phenotypes: the early onset phenotype is typically referred to as Wolman Disease and the late onset phenotype is frequently known as Cholesteryl Ester Storage Disease (CESD) [1]. Early onset LAL deficiency typically presents in the first 6 months of life and is the most rapidly fatal presentation. Growth failure associated with malabsorption, massive hepatosplenomegaly, rapidly progressive liver dysfunction, and anaemia are the predominant clinical features and key contributors to the early mortality. Survival beyond 1 year of age is highly unusual for these patients. Late onset LAL deficiency is an underappreciated cause of cirrhosis in both children and adults, and is more heterogeneous with respect to age of diagnosis. Elevation of serum transaminases, hepatomegaly, and dyslipidemia (high LDL, high triglycerides, and low HDL) are the important abnormalities. Disease complications include hepatic fibrosis with progression to cirrhosis and accelerated atherosclerosis [2], [3], [4], [5]. Diagnosis of late onset disease requires a high index of clinical suspicion as the combination of elevated transaminases, fatty liver, and dyslipidemia is also seen in patients with the much more common diagnosis of metabolic syndrome. In contrast to steatosis in patients with metabolic syndrome, which is primarily due to increased accumulations of TG, steatosis in patients with LAL deficiency is associated with increases in both CE and TG.

Monitoring liver lipid accumulation in patients has traditionally involved analysis of biopsy samples. This approach is invasive, carries an associated risk to the patient, and samples only a small region of the organ and thus may be prone to sampling errors. Magnetic resonance techniques can provide an alternative to biopsy, offering a non-invasive, safe, and repeatable method to measure liver lipid content and composition. MRI and ^1^H magnetic resonance (MR) spectroscopy methods are widely used in research studies to measure hepatic lipid content, providing either a measurement from a defined volume in the liver (via PRESS spectroscopy [6]) or an image of lipid distribution (via “Dixon method” imaging [7]). A good correlation has been observed between ^1^H MR measures of lipid content and biopsy for steatosis [8], [9]. ^13^C MR spectroscopy can also be used, and has the advantage of providing more detailed information on the chemical composition of hepatic lipids than obtained in ^1^H spectroscopy [10]. In this study, we have employed ^1^H spectroscopy to quantify and characterize the hepatic lipid signature associated with a rare genetic defect of lipid metabolism in a cohort of patients with LAL deficiency, and also utilized a biochemical assay and ^13^C spectroscopy in the preclinical studies to confirm that the observed changes in ^1^H spectra originate from cholesterol/cholesteryl ester accumulation. The previously described rat model of LAL deficiency develops liver abnormalities that closely resemble the changes seen in patients with both early and late onset LAL deficiency [11], showing CE and TG accumulation in the liver, leading to rapid development of fibrosis in this model and in other organs.

We hypothesized that the differences in lipid content and composition resulting from LAL deficiency could be detected non-invasively by MR spectroscopy, both in humans and in the preclinical model. We hypothesised that a signature of LAL deficiency could be identified (comprising detection of the increases in hepatic CE and TG content), and that the reduction in hepatic CE and TG content, following enzyme replacement therapy with sebelipase alfa (Synageva BioPharma Corp., Lexington, MA, USA), could be observed with these methods.

In addition to providing a method to monitor the efficacy of therapies that target CE accumulation, the identification and non-invasive quantification of hepatic CE content may allow for the identification of patients with fatty liver who have increased CE content and may warrant consideration for diagnostic testing for LAL deficiency. Furthermore, a non-invasive method that allows quantification of specific lipid species could help differentiate patients with LAL deficiency from patients with non-alcoholic fatty liver disease (NAFLD) due to metabolic syndrome. With the recent progression of sebelipase alfa into clinical studies for patients with LAL deficiency [12], [13], [14], such an approach would also allow the non-invasive assessment of the effects of enzyme replacement on lipid substrate accumulation in key tissues including the liver in this disease.

## Materials and methods

### Recruitment of human subjects

Study participants (n = 3) were patients with late onset LAL deficiency (also known as Cholesteryl Ester Storage Disease) enrolled into the LAL-2-NH01 substudy in the UK [15]. This is a multicenter, prospective, observational study of a subset of patients with late onset LAL to characterize clinical phenotype and disease progression. The patients’ diagnosis was confirmed by a previous diagnostic test (i.e., documented decreased LAL activity relative to the normal range of the lab performing the assay or molecular genetic testing confirming mutations consistent with the diagnosis of LAL deficiency).

A comparator group of ^1^H spectroscopy datasets from NAFLD patients was obtained from a previous ^1^H MR spectroscopy study performed at Newcastle University, where patients with NAFLD (defined as >5% intrahepatic lipid on ^1^H-MRS, n = 5) were recruited from hepatology clinics within the Newcastle upon Tyne Hospitals Foundation Trust.

### Human magnetic resonance spectroscopy

MR data were acquired on a Philips 3T Achieva whole body scanner (Philips Medical Systems, Best, The Netherlands) using a Philips 6-channel cardiac coil for ^1^H imaging and spectroscopy. ^1^H spectroscopy comprised acquisition of PRESS-localised spectra at six echo times (TR = 2.8 s, TE = 36, 50, 75, 100, 125, and 150 ms, spectral width = 2 kHz, 2k data points) from a 3 × 3 × 3 cm voxel positioned in the liver to avoid large vessels.

### Analysis of human MR spectroscopy data

Spectra were processed using the Java-based magnetic resonance user interface (jMRUI version 4.0) [16], [17] using the AMARES non-linear least square fitting algorithm [18] to determine peak areas. Resonances at 4.7, 1.3, and 0.9 ppm in ^1^H spectra were quantified, which corresponded principally to protons in water, to CH_2_ protons, and to CH_3_ protons, respectively. Initial attempts at determining T_2_ of protons contributing to these peaks highlighted significant J-coupling effects in lipid proton resonances at longer echo times. Thus no attempt was made to correct for T_2_ differences between the resonances, and reported data originate from the shortest echo time employed (36 ms).

Tissue fat fraction (v/v) calculations were performed using the widely-employed method described by Longo *et al.* [8]. Since this approach does not distinguish between cholesterol and triglyceride, and is based on the assumption that hepatic lipid has a normal (predominantly triglyceride) composition, a spectral modelling approach was also employed to provide a quantitative measure of hepatic cholesterol and fatty acid moiety content. The ratio of amplitudes of 1.3–0.9 ppm ^1^H spectral resonances was used to determine the molar ratio of cholesteryl to fatty acid moieties for each subject, and the ratio of 4.7–1.3 ppm resonances employed to quantify lipid content relative to water content. The model assumed that the hepatic triglyceride composition, and thus its *in vivo* ^1^H spectrum, was identical to that observed experimentally and described in model form by Hamilton *et al.* [19] (mean fatty acid chain length of 17.45 carbons, mean double bond content of 1.92 per fatty acid moiety, and mean number of methylene-interrupted double bonds of 0.32 per fatty acid moiety). The *in vivo* spectrum of cholesterol moieties was assumed to be identical to the National Institute of Advanced Industrial Science and Technology (Japan) database cholesterol ^1^H spectrum [20]. Thus in the spectral model, triglyceride protons contribute to 1.3–0.9 ppm ^1^H spectral resonances at a ratio of 8.0:1 (as determined by Hamilton *et al.* [19]) and cholesterol moieties contribute to these peaks at a ratio of 1:12.4. Molar ratio of fatty acid and cholesterol moieties was determined from the experimentally measured ratio of 1.3–0.9 ppm peak amplitudes for each subject and then absolute concentrations of these components calculated using the liver tissue water fraction, water and lipid proton density, and tissue density assumptions described by Longo *et al.* [8].

### Preclinical models of LAL deficiency and NAFLD

The rat model of LAL deficiency is a Donryu rat strain with a spontaneous deletion at the 3′ end of the *LIPA* gene, leading to deletion of 29 C-terminal amino acids [21]. Rats were obtained through the National Bio Resource Project for the Rat in Japan and maintained by breeding of heterozygous rats with the generation of homozygous LAL deficient progeny. Genotyping was performed by PCR analysis of DNA isolated from tail-clippings. 20–100 ng of CHELEX-extracted DNA [22] was subjected to PCR using three primers: For1, 5′-cagcacagggcacacacataggca-3′; Rev1, 5′-ccacccttgtacagcataaaagac-3′; and For2, 5′-cagagacgcaggcacaataactcc-3′ for 33 cycles of: 45 s at 95 °C, 45 s at 66 °C, 1 min 30 s at 72 °C. Reactions were resolved on ethidium bromide-stained 1% agarose gels and alleles identified by amplicons size; LAL+, 578 bp; LAL−, 341 bp.

LAL deficient rats were housed at the Animal Resources Complex in the College of Veterinary Medicine, University of Georgia, Athens, GA, USA, under the supervision of the University of Georgia Institutional Animal Care and Use Committee. Rats were provided feed (Purina Lab Irradiated Rodent Chow #5053) and water *ad libitum*. The three experimental groups comprised wild type (LAL+/+), LAL deficient (LAL−/−) genotypes, and LAL deficient animals that had received enzyme replacement therapy with sebelipase alfa. Rats were euthanized by CO_2_ inhalation at 56 days of age. Harvested livers were frozen at −80 °C for subsequent analysis by MR spectroscopy or enzymatic assay of total cholesterol and triglyceride content.

A rat model of NAFLD was employed for comparison with the LAL deficient rat liver, and was based on the model described by Kohli *et al.* [23]. Male Sprague Dawley rats were housed at Newcastle University and provided with a high fat, high carbohydrate diet for 8 weeks from an age of 5 weeks. Feed comprising 60% kcal from fat (TestDiet 58R2, IPS Product Supplies Limited, London, UK) was supplied *ad libitum*, and drinking water was supplemented with 128 fructose mM and 55 mM sucrose. Mice were euthanized and harvested livers frozen at −80 °C for subsequent MR analysis.

### Preclinical lysosomal acid lipase enzyme replacement therapy

LAL−/− rats in the enzyme replacement therapy group received four weekly doses of sebelipase alfa or vehicle from 4 weeks of age. Prior to dosing, rats were pretreated with diphenhydramine (i/p, 5 mg kg^−1^). Animals were then anesthetized with isoflurane and sebelipase alfa (0.2 mg/ml in 0.9% saline) administered intravenously via the tail vein at a dose of 3 mg kg^−1^.

### Preclinical magnetic resonance spectroscopy

All *ex vivo* MR spectra were acquired on a Varian 7T magnet and spectrometer (DirectDrive system, Varian Inc, Palo Alto, CA) at Newcastle University. Frozen liver samples (n = 6 for each group except NAFLD, where n = 3) were thawed and warmed to 37 °C, placed in 15 mm diameter plastic tubes and positioned within a 25 mm diameter birdcage coil (Rapid Biomedical, Rimpar, Germany) tuned to the ^1^H resonant frequency. Volume-localised ^1^H spectra were acquired from a 15 × 15 × 15 mm voxel using a PRESS pulse sequence employing echo times of 15 and 30 ms (TR = 10 s, spectral width = 4 kHz, 4096 data points, 4 averages). For collection of ^13^C spectra, liver samples were wrapped in thin plastic film and placed directly upon a custom in-house ^13^C circular surface coil (15 mm diameter) that was positioned above a 25 × 55 mm butterfly ^1^H decouple coil. Natural abundance ^13^C spectra were collected using a pulse-acquire sequence with ^1^H WALTZ decoupling (nominal tip = 90°, spectral width = 10 kHz, repetition time = 1.5 s, 1024 data points, 1000 averages).

### Preclinical MR data analysis

^1^H datasets were processed using jMRUI 4.0 [24] using the AMARES non-linear least square fitting algorithm [18] to determine peak areas of resonances at 4.7, 1.3, and 0.9 ppm, the mean T_2_ determined for each peak in each experimental group by fitting a monoexponential function to the data. Signal amplitude at an effective echo time of zero was determined, and these amplitudes analysed as described for human studies to determine the relative molar ratio and the absolute concentration of fatty acid and cholesterol moieties. Statistical significance of differences in liver lipid and cholesterol concentration was assessed by one-way ANOVA with *post hoc* Tukey multiple comparisons.

### Biochemical assay of rat liver lipid content

Lipids were extracted from liver samples by grinding approximately 30 mg of tissue in 600 μl of a 7:11:0.1 mixture of chloroform, isopropyl alcohol, and Igepal CA-630. Samples were centrifuged, the supernatant collected and air dried at 50 °C, then placed under vacuum for 30 min to remove the remaining solvent. The remaining solids were resuspended in a solution of 1% Igepal CA-630 in 0.9% saline (w/v) and assayed for cholesterol content using a fluorimetric cholesterol assay kit (Cayman Chemical, Ann Arbor, MI, USA) at dilutions 1:10, 1:100, and 1:1000 according to the manufacturer’s instructions. Tissue cholesterol content was determined from sample dilutions that resulted in fluorescence measurements within the assay kit standard curve.

## Results

### Human studies

**Fig. 1:**
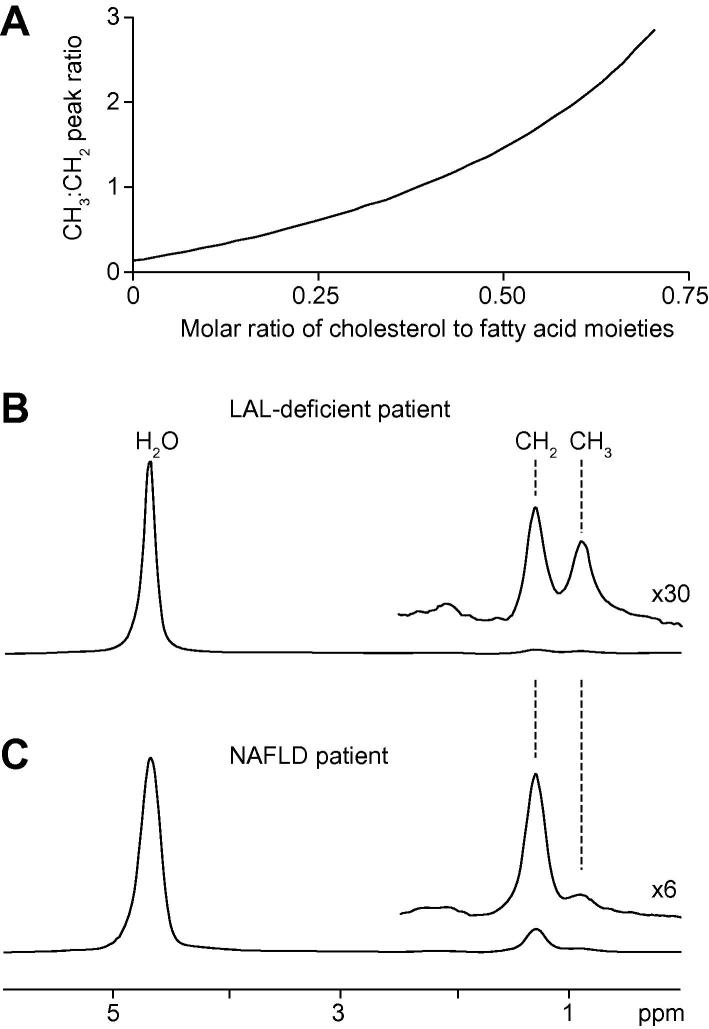
**Simulation data from spectral modelling and representative volume localised**^1^**H MR spectra acquired from study subjects.** (A) Plot of the relationship between the molar ratio of cholesterol to fatty acid moieties and the ratio of CH_3_:CH_2_ peak amplitude. (B) ^1^H spectrum from a LAL deficient subject; (C) ^1^H spectrum from a NAFLD subject. Signal from water protons at 4.7 ppm dominates the ^1^H spectra, and resonances from lipid CH_2_ and lipid and cholesterol CH_3_ resonances are seen at 1.3 and 0.9 ppm, respectively. An elevation in the ratio of CH_3_ to CH_2_ peak magnitude is observed in spectra from LAL deficient patients due to accumulation of hepatic cholesterol ester.

Fig. 1 shows spectral modelling simulation data and representative human hepatic ^1^H spectra. Fig. 1A shows data from simulation of mixtures of triglyceride and cholesterol, illustrating the change in the ratio of 1.3:0.9 ppm peak resonance amplitudes as molar fractional ratio of cholesterol increases. Cholesterol moieties contain five methyl groups with ^1^H resonances between 0.7 and 1.1 ppm (originating from the 18, 19, 21, 26, and 27 position carbons of cholesterol), and thus gives a strong resonance in this region of the ^1^H spectrum compared to a fatty acid moiety, which contains one methyl group per molecule.

Fig. 1B and C show representative ^1^H spectra acquired from a patient with LAL deficiency and from a patient with NAFLD. The spectra are dominated by signal from water at 4.7 ppm, and lipid and cholesterol CH_2_ and CH_3_ proton resonances are clearly seen at 1.3 and 0.9 ppm, respectively. The ratio of peak areas for the 1.3 and 0.9 ppm resonances in NAFLD subjects is 0.11 ± 0.05:1, in good agreement with the expected ratio of CH_2_:CH_3_ content in hepatic ^1^H spectra [19] and reflecting a lipid composition dominated by triglycerides [19]. In contrast, ^1^H spectra from LAL deficient subjects show a mean ratio of these peak areas of 0.69 ± 0.11:1, reflecting marked elevation of signal from cholesterol moieties. Table 1 shows cholesterol and fatty acid moiety concentrations as determined from the three LAL deficient and five NAFLD subjects. LAL deficient patients had a mean cholesterol moiety concentration of 21.1 ± 13.6 mM, and a mean fatty acid moiety concentration of 51.2 ± 19.2 mM. This contrasts sharply with NAFLD subjects, who had a mean fatty acid moiety concentration of 442 ± 164 mM (corresponding to a triglyceride content of 147 ± 55 mM). There is a marked difference in ratio of cholesterol to fatty acid moiety content between the groups, cholesterol was detected in only two of the five NAFLD patients and in those the content was small relative to the fatty acid moiety content. It is likely that small differences between the measured CH_3_:CH_2_ ratio and that predicted by the spectral model for 100% triglyceride content may account for the calculated cholesterol content in the NAFLD group. The differences between subject groups were statistically significant for both cholesterol and fatty acid moiety content (Mann-Whitney *U* test, *p* <0.05).

**Table 1:** **Hepatic lipid content and cholesterol and fatty acid moiety concentration measurements determined for the LAL deficient and NAFLD cohort subjects.** An elevation in the ratio of cholesterol to fatty acid moiety content is observed in LAL deficient compared to NAFLD patients.

The strong 0.9 ppm signal arising from cholesterol moieties facilitates detection of CE by ^1^H spectroscopy, and the markedly different and characteristic pattern of lipid proton resonances in the ^1^H spectrum from LAL deficient subjects compared to the NAFLD group represents a biomarker with potential for non-invasive diagnosis and monitoring of disease progression.

### Preclinical studies

**Fig. 2:**
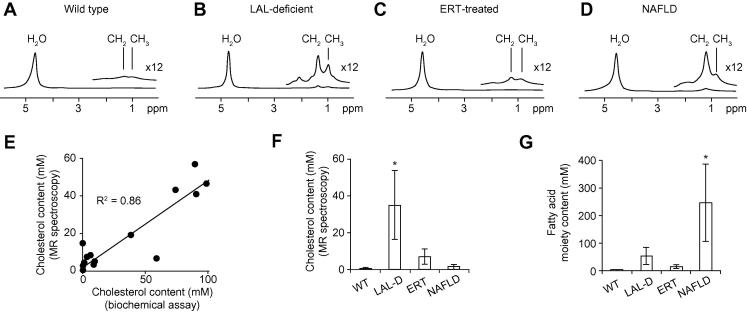
^1^**H MR spectra acquired from *ex vivo* rat liver samples from four experimental groups, and cholesterol and fatty acid moiety content measurements.** (A) Wild type hepatic ^1^H spectrum; (B) LAL deficient hepatic ^1^H spectrum; (C) sebelipase alfa-treated LAL deficient hepatic ^1^H spectrum; (D) NAFLD hepatic ^1^H spectrum; (E) correlation between biochemical assay and MR measurement of hepatic cholesterol content (R^2^ = 0.86); (F) hepatic cholesterol content as determined from MR measurements from the four experimental groups; (G) hepatic fatty acid moiety content as determined from MR measurements from the four experimental groups. * Denotes a statistically significant difference compared to the other three groups (*p* <0.05). Elevated lipid resonances are observed in spectra from LAL deficient and NAFLD rats compared to wild type, and sebelipase α-treated rats exhibit lower lipid content than their untreated counterparts. Elevation of CH_3_ to CH_2_ peak magnitude is observed in spectra from LAL deficient rats compared to NAFLD rats due to accumulation of hepatic cholesteryl ester.

Fig. 2 shows ^1^H spectra acquired from the four preclinical experimental groups (wild type, LAL deficient, sebelipase alfa-treated LAL deficient rat liver, and a rat model of NAFLD), hepatic cholesterol and fatty acid moiety concentration measurements (as determined from the MR measurements) and a plot showing correlation between MR and biochemical assay measurements of hepatic cholesterol / cholesteryl ester content. As observed in human ^1^H spectra, the preclinical MR spectra show resonances from water protons (4.7 ppm) and lipid CH_2_ (1.3 ppm) and CH_3_ (0.9 ppm) protons, and the ratio of CH_3_ to CH_2_ proton resonances differs between LAL deficient and NAFLD groups. Lipid resonances are larger in LAL deficient and NALFD samples than in the wild type group. ERT-treated LAL deficient rats show smaller CH_2_ and CH_3_ resonances than their untreated counterparts, similar to that of wild type rats.

Fig. 2E shows that a good correlation (R^2^ = 0.86) was observed between biochemical assay and MR measurements of hepatic cholesterol content, although absolute quantitation differed by a factor of approximately two. We attribute this difference to potential inaccuracies in the standard assumptions made in quantitation of spectroscopy data [8] and/or to imperfect calibration of the biochemical assay kit.

As shown in Fig. 2F and G, LAL deficient rat liver shows a marked elevation of hepatic cholesterol / cholesteryl ester content (34.9 ± 18.6 mM compared to 0.8 ± 0.4 mM in wild type), and also an increase in fatty acid moiety content (53.5 ± 30.6 mM compared to 2.1 ± 2.4 mM in wild type) that we expect to be principally due to accumulation of cholesteryl esters. In comparison, the NAFLD group shows elevated fatty acid moiety content (245 ± 139 mM) due to accumulation of triglycerides, but little elevation in cholesterol content (1.4 ± 1.3 mM). The differences between LAL deficient and NAFLD data mirror the differences observed in our human studies.

Enzyme replacement therapy with sebelipase alfa resulted in reduction in both cholesteryl and fatty acid moiety content (to 6.9 ± 4.1 mM and 14.8 ± 6.4 mM, respectively) compared to untreated animals.

The fatty acid and cholesteryl moieties both contribute to the 1.3 and 0.9 ppm resonances in the ^1^H spectrum, so a resonance uniquely attributable to cholesterol is not observed. However, ^13^C spectroscopy allows identification of resonances unique to cholesteryl moieties, providing unequivocal evidence of cholesterol accumulation in these samples that supports our conclusions regarding the biochemical origins of the observed changes in ^1^H spectra.

**Fig. 3:**
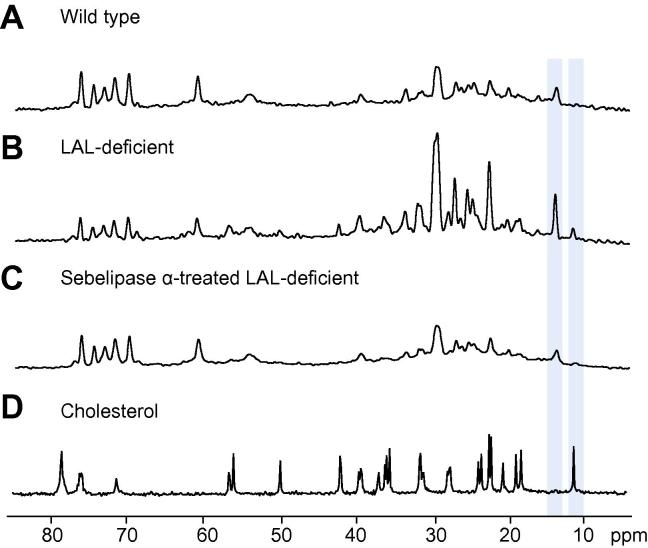
^13^**C MR spectra acquired from *ex vivo* rat liver samples, and from a solution of cholesterol in chloroform.** (A) Wild type; (B) LAL deficient; (C) sebelipase α-treated LAL deficient; (D) 1.2 M cholesterol dissolved in chloroform. Resonances from the terminal methyl group of fatty acid chains (14.1 ppm) and from the cholesterol-C18 resonance (11.8 ppm) are highlighted.

Fig. 3 shows representative ^13^C spectra from livers of wild type, LAL deficient and sebelipase alfa-treated rats, and from a solution of 1.2 M cholesterol dissolved in chloroform. Lipid resonances dominate the spectrum with the largest peak at 30 ppm corresponding to CH_2_ carbons in triglycerides, CE and phospholipids. A number of peaks in the ^13^C spectrum can be assigned as originating from the cholesterol group of CE, with the C18 resonance at 11.8 ppm (highlighted) providing a distinct and well-resolved peak separate from other resonances in the spectrum. This resonance and the resonance arising from fatty acid methyl carbons (14.1 ppm, highlighted) each originates from a single atom within their respective moieties (as opposed to the lipid CH_2_ resonance at 30 ppm, for example). Elevation of cholesteryl moiety content is observed in LAL deficient liver samples compared to wild type, and reduced in sebelipase alfa-treated liver.

**Fig. 4:**
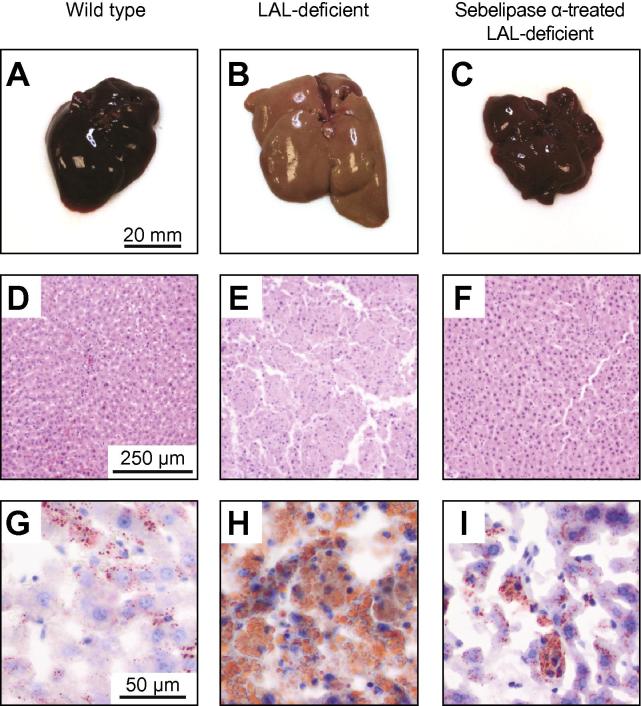
**Physical and histological appearance of liver samples from wild type, LAL deficient, and sebelipase α-treated LAL deficient rats.** (A–C) Physical appearance; (D–F) haematoxylin and eosin-stained histological sections; (G–I) Oil Red-O-stained histological sections. (This figure appears in colour on the web.)

Fig. 4A–C shows the physical appearance of excised rat livers from wild type, LAL deficient, and LAL deficient sebelipase alfa-treated groups. In contrast to the normal dark red colour of livers from wild type rats, LAL deficient livers have a pale orange/yellow colour resultant from lipid accumulation. In other experiments, we have observed that the liver appearance of LAL deficient rats is already abnormal at 4 weeks of age and becomes more marked due to the increasing lipid content in LAL deficient rats between 4 and 8 weeks of age (data not shown). Livers from LAL deficient rats treated with sebelipase alfa showed a more normal red colour that resembled the appearance of livers from wild type rats. H&E and Oil Red-O stained histological sections are shown in Fig. 4D–F and G–I, respectively. Oil Red-O stained histological sections show elevated lipid density in LAL deficient rat liver samples compared to wild type and sebelipase alfa-treated rat liver samples.

## Discussion

We have used MR spectroscopy to characterize the lipid content and composition in LAL deficient and NAFLD patients, and in livers from LAL deficient, NAFLD, wild type and sebelipase alfa-treated LAL deficient rats. We conclude that LAL deficiency is associated with distinct ^1^H and ^13^C MR signatures arising from cholesterol moieties, and that the ^1^H signature can be used to identify and quantify abnormal lipid substrate content in patients with LAL deficiency via this non-invasive method. Data from the preclinical ^1^H spectroscopy studies mirrored findings from the human ^1^H spectra: the ratio of ^1^H spectral resonances from CH_2_ and CH_3_ groups was substantially altered in LAL deficiency compared to NAFLD. Preclinical ^13^C spectroscopy and biochemical assay data provided confirmation that the alteration in lipid composition and content observed by ^1^H spectroscopy originated from the expected accumulation of CE and TG (the normal substrates for the deficient enzyme), with relative predominance of CE to TG compared to wild type, consistent with earlier studies in both patients with LAL deficiency and preclinical disease models [25], [26], [27].

^1^H spectroscopy is widely used in clinical research studies to measure tissue lipid content, including hepatic lipid content [8], and intra and extramyocellular lipid content in muscle [28]. Our preclinical results augment a previous report of mouse LAL deficiency [29] which employed ^1^H MRI for non-invasive repeated measures of fat distribution and liver volume, though this study did not differentiate between the different lipid components in the LAL deficient mouse model. A range of methods of calculating lipid content from ^1^H spectra have been reported in the literature, including ratio of CH_2_ to water proton peak intensities, correction of peak intensities for relaxation effects, and quantitation of all visible lipid peaks in the spectrum [19], [30]. Our measurement of lipid content employs spectral modelling to determine relative contributions of cholesteryl and fatty acid moieties to the hepatic lipid MR signal. A limitation of our approach is that the repetition time employed for ^1^H spectroscopy in the human studies was insufficient to allow complete T_1_ relaxation, and as a result our spectra may exhibit some T_1_ weighting. Differences in T_1_ between water and lipid components would thus have the potential to influence results of lipid content quantitation.

Based on hepatic lipid volume fraction, only one of the three LAL deficient patients studied meet the criteria of NAFLD (>5% intrahepatic lipid). Thus employing this widely-used hepatic analytical approach developed for assessing liver fat content in NAFLD could result in failure to recognise hepatic lipid accumulation in these patients. Our data thus demonstrate the importance of measuring CH_3_:CH_2_ peak ratio to assess hepatic lipid composition.

Although the calculated hepatic cholesterol content was markedly elevated in the LAL deficient patients compared to NAFLD, the levels were lower than the levels seen in the preclinical disease model. This most likely reflects differences in disease severity between the rat model and these LAL deficiency patients, though it is also possible that not all CH_2_ and CH_3_ protons were visible in the MR spectra. Liver samples used in the preclinical studies exhibited smaller proton CH_2_ and CH_3_ resonances at room temperature after thawing from frozen, compared to when scanned after warming to 37 °C then cooled to room temperature (data not shown), illustrating the influence of lipid molecule mobility on MR visibility. Further studies are required to investigate whether there is potential for visibility to be affected *in vivo* resulting from low lipid molecule mobility arising from the unusual lipid composition and intracellular distribution associated with LAL deficiency.

Our ^13^C preclinical data demonstrate an alternative measure of hepatic cholesterol content via a resonance unique to cholesterol moieties, but our attempts at translation to human studies yielded spectra dominated by resonances from subcutaneous lipid signals, masking any small ^13^C resonances from cholesterol (data not shown). It is possible that ^13^C spectroscopy with improved spatial localisation may allow detection of such resonances in humans, though the ^1^H spectroscopy approach is several orders of magnitude more sensitive than the ^13^C measurements of hepatic CE due to the higher sensitivity of ^1^H measurements, the 100% isotopic natural abundance of ^1^H (compared to 1.1% for ^13^C), and the large number of methyl protons contributing to the ^1^H CE signal at 0.9 ppm.

A good correlation was observed between MR and biochemical assay measurements of hepatic cholesterol content, and the large relative differences in cholesterol and TG content observed between LAL deficiency and NAFLD (human) or wild type (preclinical) data underscore the potential of this approach for clinical diagnosis or treatment monitoring.

Our enzyme replacement therapy data demonstrate reduction of CE and TG substrate after treatment with sebelipase alfa. We have previously established that sebelipase alfa corrects a number of clinically relevant abnormalities including growth failure, hepatosplenomegaly, and transaminase elevations in the LAL deficiency disease model [27], [31]. The MR data demonstrates that these improvements are associated with reduction in tissue CE and TG content. The potential for ^1^H MR spectroscopy as a tool for treatment monitoring in humans is apparent, providing a non-invasive and repeatable measure of hepatic TG and CE content capable of being performed on the majority of clinical MR scanners (unlike ^13^C spectroscopy, which requires additional hardware and protocol development).

Our findings provide insights that may be applied to the identification of increased cholesteryl ester content in patients with fatty liver which either represent undiagnosed cases of late onset LAL deficiency or may be of interest as a potential covariate impacting the risk of disease progression (given recent observations from disease models on the effects of increasing hepatic cholesterol content on fibrosis progression [32], [33]). Such an approach demonstrates the value of MR spectroscopy as it allows repeatable, non-invasive measurement of the concentration of a metabolite central to the progression of a disease. Our findings from this preclinical and human study thus demonstrate the potential utility of our methods for monitoring of disease pathophysiology and response to treatment using a novel diagnostic biomarker.

## Financial support

This work was supported by Synageva BioPharma Corp, Lexington, MA, USA and by grant G0801239 from the Medical Research Council, UK (P.E.T.), by the ‘Fatty Liver Inhibition of Progression’ (FLIP) project funded by the European Union Seventh Framework Programme (FP7/2007-2013) under grant agreement Health-F2-2009-241762, and by the National Institute for Health Research Biomedical Research Centre on Ageing & Age Related Diseases.

## Conflict of interest

MCL, DC, WH, JVR and AGQ are full time employees and stockholders of Synageva Biopharma Corp.
